# The anti-apoptotic and prognostic value of fibroblast growth factor 9 in gastric cancer

**DOI:** 10.18632/oncotarget.9131

**Published:** 2016-05-02

**Authors:** Chuanli Ren, Hui Chen, Chongxu Han, Deyuan Fu, Fuan Wang, Daxin Wang, Li Ma, Lin Zhou, Dongsheng Han

**Affiliations:** ^1^ Clinical Medical Testing Laboratory, Northern Jiangsu People's Hospital and Clinical Medical College of Yangzhou University, Yangzhou, China; ^2^ Geriatric Medicine, Northern Jiangsu People's Hospital and Clinical Medical College of Yangzhou University, Yangzhou, China; ^3^ Breast Oncology Surgery, Northern Jiangsu People's Hospital and Clinical Medical College of Yangzhou University, Yangzhou, China; ^4^ Department of Interventional Radiography, Northern Jiangsu People's Hospital and Clinical Medical College of Yangzhou University, Yangzhou, China; ^5^ Laboratory of Hematology, Northern Jiangsu People's Hospital and Clinical Medical College of Yangzhou University, Yangzhou, China; ^6^ Department of Epidemiology and Biostatistics, Ministry of Education (MOE) Key Laboratory of Modern Toxicology, School of Public Health, Nanjing Medical University, Nanjing, China

**Keywords:** gastric cancer, FGF9, apoptosis, prognosis

## Abstract

Fibroblast growth factor (FGF) 9 is a member of the FGF family, which promotes carcinogenesis in some solid tumours. However, its biological and prognostic significance in gastric cancer (GC) is unclear. We examined FGF9 expression in 180 GC and corresponding non-tumorous gastric tissue samples by immunohistochemistry and evaluated its role in predicting tumour prognosis. Knockdown of FGF9 by siRNA inhibited cell growth and induced apoptosis in GC cell lines. Fifty of the 180 GC specimens (27.8%) had high FGF9 protein expression, whereas decreased or unchanged expression was observed in 130 cases (72.2%). High FGF9 expression was a significant predictor of poor survival (28.1 vs. 55.8 months, *P* < 0.001). After stratification according to AJCC stage, FGF9 remained a significant predictor of shorter survival in stage II (30.6 vs. 64.9 months, *P* < 0.001) and stage III GC (29.7 vs. 58.9 months, *P* < 0.001). Multivariate and univariate analysis showed that higher expression of FGF9 can be used as a predictor for poor prognosis (HR, 2.95; 95% CI, 1.97–4.41; *P* < 0.001; and HR, 2.94; 95% CI, 2.01–4.31; *P* < 0.001, respectively). FGF9 may provide the anti-apoptotic function and be useful as a novel independent marker for evaluating GC prognosis

## INTRODUCTION

Gastric cancer (GC) accounts for high morbidity and mortality globally [1]. Almost half the worldwide GC incidence occurs in China [1, 2]. GC initiation is a multistep and multiple gene mutation process, and phenotypic and genetic characterization of GC will provide a new way to develop tailored therapies [3, 4]. Studies have shown that different molecular expression profiles in GC may have different prognoses [2, 5]. Four molecular subtypes of GC were tied to molecular alterations, disease progression and prognosis [3, 5]. However, the molecular mechanisms for GC development and prognosis is still unclear [2].

FGF (Fibroblast growth factors) family includes 23 family members with key functions in cellular proliferation, survival, migration, differentiation, tumourigenesis and other function [6, 7]. Among the FGFs, only 18 are ligands for FGF receptors (FGFRs), and these ligands bind FGFRs to induce downstream signalling [6]. FGF binding to FGFR promotes the change of FGFR structure, resulting in developmental signalling pathway activation, which are responsible for many normal biological functions [6, 8–10]

FGFs signalling occurs through four main signal pathways to maintain its physiological function [6, 8–10]. Notably, FGFR2 is preferentially amplified in the diffuse type of GC and may be a therapeutic target in GC [11]. Several studies have linked dysregulated FGF9 in various cancers. High expression of FGF9 in lung cancer was identified as a novel unfavourable prognostic indicator [10]. Furthermore, a previous study showed that miR-26a inhibited GC development by targeting FGF9 [12].

However, the molecular significance of FGF9 and its prognosis value in GC has not been fully elucidated. This study was to examine the effects of FGF9 on the proliferation and apoptosis of GC and to evaluate the FGF9 expression and its prognosis value in a Chinese population of GC patients.

## RESULTS

### Knocking down FGF9 inhibits growth and induces apoptosis in GC cells

**Figure 1:**
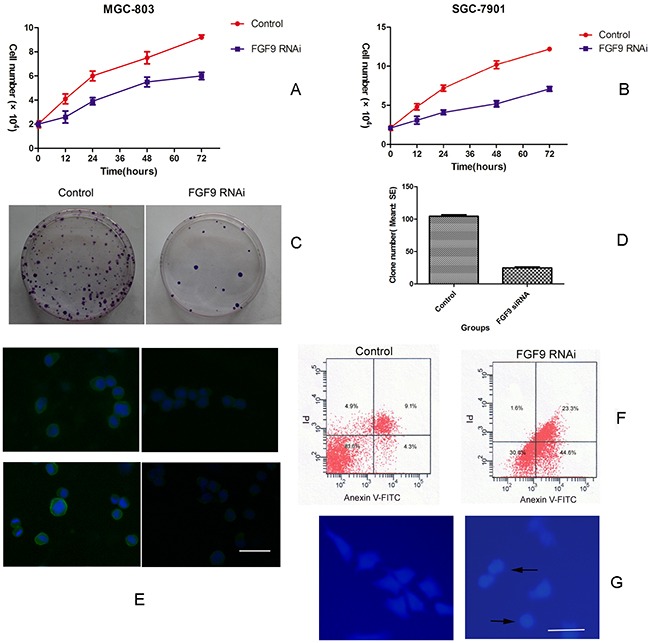
Downregulation of FGF9 by siRNA in GC cells inhibits cell growth and induces apoptosis Growth of MGC-803 **A.** and SGC-7901 cells **B.** transfected with FGF9 siRNA or control. MGC-803 cells transfected with FGF9 siRNA or control were analysed by colony formation assay **C.** MGC-803 cells transfected with FGF9 siRNA or control were analysed by a histogram (*P < 0.001) **D.** MGC-803 (upper layer) and SGC-7901 (below layer) cells were transfected with FGF9 siRNA (right figure) or control (left figure) and the expression of FGF9 was detected by immunofluorescence staining **E.** MGC-803 cells were transfected with FGF9 siRNA or control and apoptotic cells were evaluated by Annexin V-FITC and PI staining and FACS **F.** Apoptotic morphological analysis of MGC-803 cells transfected with FGF9 siRNA or control by DAPI staining **G.** All data are presented as mean±s.e.m from at least three separate experiments.

To first examine the function of FGF9 in GC cells, FGF9 siRNA or control siRNA were transfected into two GC cell lines, MGC-803 and SGC-7901, and the effects on growth and apoptosis were evaluated. Cell growth and colony formation experiments showed that knockdown of FGF9 inhibited cell growth in both gastric cancer cell lines compared with control siRNA transfections (*P*<0.01)(Figure 1A, 1B, 1C). DAPI staining and flow cytometry analysis showed that knockdown of FGF9 induced apoptosis in gastric cancer cells compared with controls (Figure 1D, 1F). Immunofluorescence staining showed that MGC-803 (Figure 1E) and SGC-7901 (Figure not shown) were overexpression of FGF9. After the tranfection of FGF9 for 24 hours, the expression of FGF9 was decreased obviously (Figure 1E).

Together these results demonstrate that knocking down FGF9 inhibits cell growth and promotes apoptosis in GC cell lines.

### FGF9 expression in GC and paracancerous tissues

**Figure 2:**
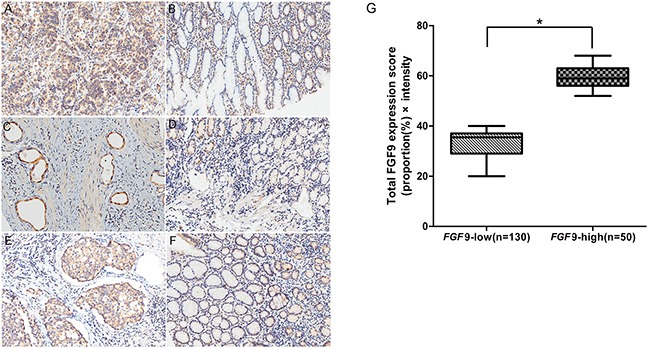
Immunohistochemical analysis of FGF9 expression and survival curves in patients with GC according to FGF9 levels **A.** High FGF9 expression in gastric adenocarcinoma and low expression in corresponding non-cancerous gastric tissues **B.** High FGF9 expression in gastric adenocarcinoma, part of signet-ring cell carcinoma and low expression in corresponding non-cancerous gastric tissues **C.** and low FGF9 expression in corresponding non-cancerous gastric tissues **D.** High FGF9 expression in gastric tubular adenocarcinoma **E.** and low FGF9 expression in corresponding non-cancerous tissues **F.** The total FGF9 expression score was calculated by multiplying the proportion (%) of cells expressing FGF9 with the intensity score described in Methods. The thick line indicates the median score in each group **G.** *P < 0.001, FGF9-low group vs. FGF9-high group (Mann–Whitney U-test).

Next we examined the expression of FGF9 in 180 GC and its corresponding normal gastric tissues by immunohistochemistry staining. In GC and normal paracancerous tissues, FGF9 was located in the cytoplasm. (Figure 2). Among the 180 total GC samples, FGF9 expression was decreased or unchanged in 72.2% of the GC cases (130/180) and increased in 27.8% (50/180) compared with the normal paracancerous tissues.

### Relationship between FGF9 and its clinicopathological parameters in GC

Next we examined the relationships between FGF9 levels and clinicopathological parameters of GC patients (listed in Table 1). We observed a tendency between age and FGF9 expression levels in GC patients (χ^2^ = 5.634, *P* = 0.018), but no significance was found between FGF9 expression level and other clinicopathological variables, including gender, site, TNM, tumour size, nodal status, metastasis status and tumour invasion (Table 2).

**Table 1:** Characteristics of the study subjects

Clinicopathologic features	Number	Percentage (%)
**Age (years)**		
**<60**	62	34.4
**≥60**	118	65.6
**Gender**		
**male**	130	72.2
**female**	50	17.8
**Tumour Size (cm)**		
**<10**	156	86.7
**≥10**	24	23.3
**Tumour site**		
**cardia**	26	14.4
**Non-cardia**	154	85.6
**Pathological type**		
**adenocarcinoma**	176	97.8
**undifferentiated carcinoma**	4	2.2
**Tumour status**		
**T1+T2**	24	13.4
**T3+T4**	155	86.6
**Nodal status**		
**negative**	45	25.0
**positive**	135	75.0
**Metastasis status**		
**M0**	166	92.2
**M1**	14	7.8
**Tumour stage**		
**I**	17	9.4
**II**	56	31.1
**III**	92	51.1
**IV**	14	7.8
**Follow-up time (months)**	79.2-97.2	
**Prognosis**		
**alive**	49	25.8
**dead**	125	74.2
**patients lived for ≥5 years**	74	41.1
**patients lived for <5 years**	106	58.9

**Table 2:** FGF9 expression and clinicopathological features in patients with gastric adenocarcinoma

Characteristics	FGF9 low or unchanged (%)	FGF9 high (%)	χ2 or Fisher's exact test	*P*-value
**Age (years)**			5.634	0.018
**<60**	38(29.2)	24(48.0)		
**≥60**	92(70.8)	26(52.0)		
**Gender**				0.103
**male**	89(68.5)	41(82.0)	2.659	
**female**	41(31.5)	9(18.0)		
**Local invasion**				
**T1+T2**	22(17.1)	2(4.0)		0.026
**T3+T4**	107(82.9)	48(96.0)		
**Site**			0.708	0.4
**gastric cardia**	17(13.1)	9(18.0)		
**non-cardia**	113(86.9)	41(82.0)		
**TNM stage**				
**I+II**	19(14.7)	5(10.0)	0.694	0.405
**III+IV**	110(85.3)	45(90.0)		
**Nodal status**			2.991	0.084
**positive**	37(28.5)	8(16.0)		
**negative**	3(71.5)	42(84.0)		
**Distant metastasis**				0.760
**M0**	119(96.5)	47(94.0)		
**M1**	11(3.5)	3(6.0)		
**Tumour size(cm)**				0.139
**≥10**	20(15.5)	4(8.0)		
**<10**	109(84.5)	46(92.0)		

### Survival analysis

The median overall survival (OS) in our study was 41 months as we reported before [13]. High FGF9, tumour size, stage, tumour status, node status, and distant metastasis were significantly poor prognostic parameters for OS in GC patients (*P* < 0.001, *P* < 0.001, *P* = 0.008, *P* < 0.001, *P* = 0.001, and *P* = 0.005). Other predictors, such as age, gender, and location, were not linked with prognosis significantly (*P* = 0.005, Table 3).

**Table 3:** Univariate analysis of survival in patients with GC

Variable	Mean survival time month (±SE)	95% CI (Month)	*P*
**Age (years)**			0.132
**<60**	57.3(4.8)	47.9-66.6	
**≥60**	48.9(3.4)	42.3-55.5	
**Gender**			0.668
**Male**	52.5(3.3)	46.0-59.0	
**Female**	50.5(5.1)	40.6-60.4	
**Tumour site**			0.986
**Gastric cardia**	53.0(7.4)	38.5-67.4	
**Non-cardia**	51.8(3.0)	45.9-57.7	
**Stage of disease**			0.000
**I–II**	70.0(4.0)	62.2-77.8	
**III–IV**	39.4(3.4)	32.8-46.1	
**Tumour status (p)**			0.008
**T1−T2**	67.5(5.6)	56.5-78.4	
**T3−T4**	48.5(3.0)	42.6-54.4	
**Node status**			0.000
**Negative**	72.9(4.8)	63.6-82.3	
**Positive**	44.8(3.1)	38.7-51.0	
**Distant metastasis**			0.005
**No**	54.0(2.9)	48.2-59.7	
**Yes**	30.2(6.4)	17.6-42.8	
**FGF9**			0.000
**High expression**	28.1(3.5)	21.2-35.0	
**Low/unchanged**	55.8(3.5)	48.9-62.7	
**Tumour size (cm)**			
**≥10**	28.4(5.0)	18.5-38.2	0.001
**<10**	54.8(3.1)	48.8-60.8	

**Figure 3:**
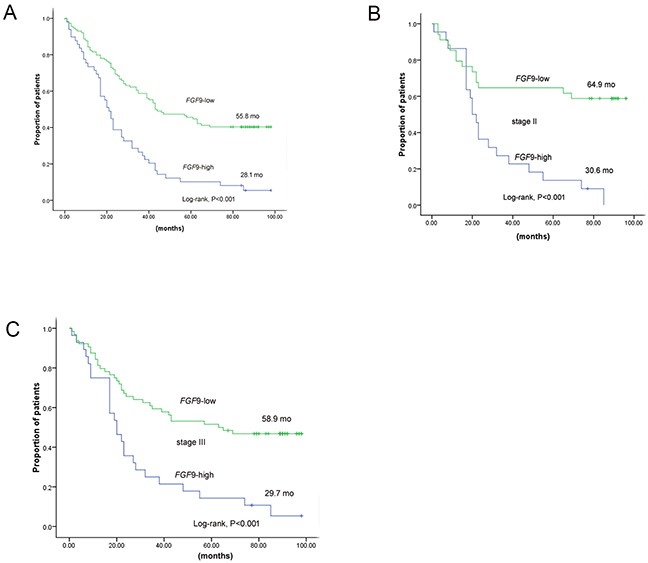
The prognosis of GC patients with high expression of FGF9 and low/unchanged expression of FGF9 **A.** Kaplan–Meier curves of 163 GC patients according to FGF9 expression. **B.** Kaplan–Meier curves of 56 GC patients according to FGF9 expression in stage II. **C.** Kaplan–Meier curves of 92 GC patients according to FGF9 expression in stage III. *P < 0.001 (log-rank test).

Higher levels of FGF9 predicted a significant parameter of poor survival compared with lower one (28.1 months vs. 55.8 months, *P* < 0.001). FGF9 with higher expression remained a notable indicator of shorter survival in stage II (30.6 months vs. 64.9 months, *P* < 0.001, n=56) and stage III (29.7 months vs. 58.9 months, *P* < 0.001, n=92) (Figure 3) by tumour stage stratification in GC.

We found these parameters predicting poor prognosis as we reported before by Multivariate Cox regression study [5]: tumour status (hazard ratio [HR], 1.77; 95% confidence interval [CI], 0.73–4.29; *P* = 0.204), stage (HR, 2.08; 95% CI, 1.15–3.77; *P* = 0.015), lymph node metastasis (HR, 1.70; 95% CI, 0.84–3.42; *P* = 0.139), high FGF9 expression (HR, 2.95; 95% CI, 1.97–4.41; *P* < 0.001) and tumour size (HR, 2.13; 95% CI, 1.30–3.47; *P* = 0.003)(Table 4). GC patients with low or unchanged FGF9 expression were correlated with better prognoses, while higher expression ones were not.

**Table 4:** Multivariate Cox regression analysis of potential prognostic factors for survival in 180 patients with GC

Variables	Univariate analysis		Multivariate analysis	
	HR(95%CI)	*P*-value	HR(95%CI)	*P*-value
**Tumour status, T1−T2 vs. T3−T4**	3.91(1.72-8.91)	0.001	1.77(0.73-4.29)	0.204
**Stage, I − II vs. III−IV**	2.83(1.88-4.28)	0.000	2.08(1.15-3.77)	0.015
**LNM, no vs. yes**	2.83(1.88-4.28)	0.000	1.70(0.84-3.42)	0.139
**Low FGF9 vs. High FGF9**	2.94(2.01-4.31)	0.000	2.95(1.97-4.41)	0.000
**Tumour size (cm), <10 vs. ≥10**	2.34(1.45-3.79)	0.002	2.13(1.30-3.47)	0.003
**Age (years), ≥60 vs. <60**	1.35(0.91–2.01)	0.137	1.66(1.09-2.53)	0.018
**Gender, male vs. female**	0.92(0.61–1.37)	0.671	1.04(0.68-1.60)	0.843
**Tumour site, gastric cardia vs. non-cardia**	1.51(1.93–2.45)	0.093	1.58(0.59-2.62)	0.077

## DISCUSSION

FGF signal play an important role in cellular function, including growth, invasion, and epithelial-to-mesenchymal transition(EMT) [14, 15]. Mutations or gene amplification of FGFR1, FGFR2, and FGFR3 have been reported in different kinds of solid cancers [16–19]. The FGF/FGFR signal has been regarded as a potential therapeutic target, drug development and prognostic markers for various kinds of solid cancers [6, 20].

FGF9 is involved in various biological processes. For example, FGF9 may inhibit osteogenesis in mesenchymal stem cells in vitro [21] and participate in the development of GC by its autocrine stimulation pattern [15]. FGF2, FGF9 and FGF10 can stimulate proliferation, treatment sensitivity, and apoptosis of lung cancer cells [7].

FGF9 activation of FGF/FGFR signals has been reported in some kinds of cancers [10]. FGF9 has been shown to be dysregulated in ovarian endometrioid adenocarcinoma [22], hepatocellular carcinoma [23], prostate carcinoma [24] and GC [12]. But the FGF9 serum concentration in lung cancer patients was too low to be detected by ELISA assay [7]. It's reported that FGF9 mRNA high expression was correlated with poor survival [10]. Moreover, miRNA-FGF9 pathway is important for lung development and regarded as an initiating factor for pleuropulmonary blastoma [25]. Induction of FGF9 in adult lung can lead to the rapid formation of epithelial tumours [26]. Overexpressing FGF9 can promote the formation of reactive stroma and initiation in prostate cancer cells [27].

A previous study showed that FGF9 from cancer-associated fibroblasts may activate invasion ability of GC cells [28]. In our study, we found that knockdown of FGF9 resulted in reduced cell growth and induced apoptosis in GC. Thus, FGF9 may play an important oncogene function in GC cells and may be a potential target for GC.

In this study, we found that 27.8% (50/180) of GC specimens had high FGF9 expression compared with normal paracancerous tissues. Our previous work found that miR-486-5p can decrease FGF9 protein expression in GC. Low levels of FGF9 in a fraction of GC patients were linked to longer overall survival [2]. So high expression of FGF9 may predict poor prognosis through aberrant regulation of miR-486-5p in patients with GC.

It's reported that miR-26a inhibits tumour growth by the target of FGF9 in GC [12]. Furthermore, the authors showed that FGF9 overexpression could protect GC cells from apoptosis induced by miR-26a. In addition, low expression of miR-26a can lead to poor survival in GC patients [12]. Together this suggests that FGF9, as the target gene of miR-26a, may promote tumour growth and inhibit GC apoptosis.

Interestingly we found a significant difference in age for GC patients with low/unchanged and high levels of FGF9 (*P* = 0.018). It indicates that GC patients of older age show a tendency for high expression of FGF9. The underlying mechanism of this phenomenon is unknown.

In our research, 180 patients with GC were evaluated for FGF9 expression and 163 were included in the OS analysis. As expected, traditional pathological parameters, such as stage (*P* < 0.001), tumour status (*P* = 0.008), node metastasis (*P* < 0.001), tumour size (*P* = 0.001), and distant metastasis (*P* = 0.005), were significantly poor prognostic parameters for OS in patients with GC. Moreover, high FGF9 expression (*P* < 0.001) was also a significantly poor prognostic predictor for OS in patients with GC. However, as the prevail of molecular and genotype heterogeneous of GC, patients with the same TNM stage may have distinct prognosis [4, 29]. So it is urgent to find an ideal tumour maker to evaluate the prognosis in individual GC patient. In our study, higher levels of FGF9 can be a significantly poor predictor survival in stage II and stage III GC (*P* < 0.001). Furthermore, multivariate and unvaried Cox analyses indicated a shorter OS with high FGF9 expression (*P* < 0.001). Together these data indicate that high level of FGF9 may be used as an independent indicator for poor prognosis in GC.

Several FGFR tyrosine kinase inhibitors are being exploited for the treatment of GC [11, 14, 30–34], although the current drug development process is challenge [20]. FGF9 may have the anti-apoptotic function and be used as a potential novel maker for prognosis evaluation in GC. However, the mechanism through which FGF9 plays a key role in GC disease process need to be clarified in future studies.

## MATERIALS AND METHODS

### Cell culture and FGF9 siRNA transfection

The GC cell lines MGC-803 and SGC-7901 were purchased from the Chinese Academy of Medical Science (Beijing, China) and maintained at 37°C in 5% CO_2_ in RPMI-1640 (MGC-803) or DMEM (SGC-7901), respectively, supplemented with 10% fetal bovine serum (FBS) with penicillin and streptomycin (Gibco BRL, NY, USA). FGF9 and control siRNAs were purchased from GenePharma (Shanghai, China) and the sequences of these siRNAs are as follows: FGF9-homo-1044 siRNA5′-CUGGAUUUCACUUAGAAAUTT-3′, 3′-AUUUCUAA GUGAAAUCCAGTT-5′; FGF9-homo-1201 siRNA 5′-GGAGCUGUAUGGAUCAGAATT-3′, 3′-UUCUGAU CCAUACAGCUCCTT-5′; FGF9-homo-1315 siRNA 5′-GCGAUACUAUGUUGC AUUATT-3′, 3′-UAAUGCA ACAUAGUAUCGCCT-5′; and control 5′-CAGUACUUU UGUGUAGUACAA-3′. Three FGF9 siRNA and controls were synthesized and the inhibition effect was evaluated byimmunofluorescence staining. Transfections were performed using Lipofectamine 2000, according to the manufacturer's instructions (Invitrogen, Carlsbad, USA).

### Immunofluorescence staining

The GC cell lines MGC-803 and SGC-7901were cultured in 12-well plates and then transfections were performed using Lipofectamine 2000. The experimental groups and controls were fixed 4% poly formaldehyde for 40 min and were performed 100ul FGF9 antibody (#ab71395, Abcam Cambridge, UK) overnight at 4°C. After washing by PBS three times, a goat anti-rabbit IgG-PE was incubate for 1 hours(sc-3739, Santa Cruz, USA) and DAPI was stained for nucleus.

### Cell proliferation assay

Cells were plated in 12-well plates at the desired cell concentrations. Cell were trypsinized and performed counting using a Coulter Counter (Beckman Coulter, Fullerton, USA) at the indicated time points in triplicate.

### Analysis of apoptosis

After FGF9 or control siRNA transfection, both attached and floating cells were harvested at different time points and washed with PBS. The fraction of apoptotic cells was determined by nuclear staining and two-colour analysis with Annexin V-PI. Nuclear morphology was assessed with DAPI staining. Briefly, cells were fixed with a solution of 3.7% formaldehyde, 0.5% NP-40, and 10 mg/mL DAPI and analysed by fluorescence microscopy. Apoptotic cells with condensed chromatin and fragmented nuclei were counted from three fields for each sample. All experiments were carried out in triplicate. For Annexin V-PI staining, the GC cells were stained using an Annexin V-PI assay kit (BioVision Co., Ltd, CA, USA) and quantified and analysed using a BD FACSCalibur flow cytometer (Becton Dickinson).

### Colony formation assay

After FGF9 or control siRNA transfection, GC cells were trypsinized and seeded in 10-cm dishes (10^4^ cells per dish) and cultured in DMEM supplemented with 10% FBS without anticancer drugs. After 14–16 days, cells were fixed in 3.7% formaldehyde and stained with 0.25% crystal violet (AMRESCO) in PBS for 30 minutes. Clones were washed with water and counted. All experiments were carried out in triplicate.

### Patients and tissue samples

Paraffin-embedded tissue samples were collected retrospectively in the Biobank Center at the National Engineering Center for Biochip at Shanghai(Shanghai Outdo Biotech Cop., Ltd, Shanghai, China). Samples from tumour tissue and corresponding neighbouring normal tissue were obtained from 180 patients with histologically diagnosed GC who underwent surgical resection between 2006 and 2008.

The following clinicopathological data were obtained from the original pathology results: age, gender tumour size, location and invasion, lymph node metastases, and tumour stage. Staging of GC was assessed according to the AJCC criteria. The clinical and pathological data for the patients is provided in Table 1. Written informed consent was obtained from all patients, and the protocol was approved by the Ethical Committee of the National Engineering Center for Biochip at Shanghai.

Follow-up times were measured from the date of surgery to the date of death for all 180 GC patients. The last follow-up point was in September 2014, and seventeen patients were out of touch in September 2014, but all had survived for five years in the previous follow up. The median follow-up time was 7.1 years (range 6.6–8.1 years). Among the 180 patients, 115 died during the follow-up period.

### Tissue microarray construction

Tissue microarrays (TMAs) were constructed using appropriate tissue cores from formalin-fixed and paraffin-embedded samples as described previously [2, 35].

### Immunohistochemistry

Immunohistochemical analysis was performed on 180 GC specimens. All tumour tissues and the surrounding gastric tissues were removed and embedded in paraffin and cut into 4-cm-thick sections. These sections were deparaffinized, rehydrated, and incubated in 0.03% H_2_O_2_ in 95% methanol at room temperature for 20 min to block endogenous peroxidase activity. Antigen retrieval was performed using water bath pretreatment (Immunosaver; Nisshin EM, Tokyo, Japan) at 98°C for 45 min. All sections were incubated for 20 min with normal horse serum to eliminate non-specific staining and incubated with anti-human FGF9 antibody (#ab71395, Abcam Cambridge, UK) overnight at 4°C. This step was followed by incubation with the secondary antibody (ImmPRESS Reagent Kit; Vector Laboratories, Burlingame, CA) for 30 min. Slides were then incubated in diaminobenzidine (DAB)/Tris solution (3DAB/Tris) tablets (Muto Pure Chemicals, Tokyo, Japan) diluted in 150 ml of distilled water supplemented with 15 μl of 30% H_2_O_2_. Finally, the slides were counterstained with haematoxylin. The proportion of cells stained and the staining intensity score were assessed by the pathologist as follows: 0, absence of staining; 1, weakly stained; 2, moderately stained; and 3, strongly stained. The total score was calculated by multiplying the proportion score with the intensity score [2, 36, 37]. High expression of FGF9 means that the expression of FGF9 is higher than that of normal tissue adjacent to cancer. Low expression of FGF9 means that the expression of FGF9 is lower than that of normal tissue adjacent to cancer.

### Statistical analysis

Associations between clinicopathological parameters and FGF9 expression were evaluated using χ^2^ tests. When sample numbers in some categorical cells were less than 5, Fisher's exact test was used. Overall survival was calculated and survival curves were obtained using the Kaplan–Meier method; differences between groups were compared using log-rank tests. Significant variables in univariate models were further analysed by multivariate Cox proportional hazards regression models. All analyses were performed using the SPSS software package (SPSS Inc., Chicago, IL, USA, version 17.0). All tests were two-sided and *P* values < 0.05 were considered statistically significant.
